# Elucidation of quantitative structural diversity of remarkable rearrangement regions, shufflons, in IncI2 plasmids

**DOI:** 10.1038/s41598-017-01082-y

**Published:** 2017-04-19

**Authors:** Tsuyoshi Sekizuka, Michiko Kawanishi, Mamoru Ohnishi, Ayaka Shima, Kengo Kato, Akifumi Yamashita, Mari Matsui, Satowa Suzuki, Makoto Kuroda

**Affiliations:** 1 Pathogen Genomics Center, National Institute of Infectious Diseases, 1-23-1 Toyama, Shinjyuku-ku, Tokyo 162-8640 Japan; 2 Assay Division II, Bacterial Assay Section, National Veterinary Assay Laboratory, Ministry of Agriculture, Forestry and Fisheries, 1-15-1 Tokura, Kokubunji-shi, 185-8511 Tokyo Japan; 3 Ohnishi Laboratory of Veterinary Microbiology, 10-3-3 Nishirokujyouminami, Shibetsugunnakashibetsu-cho, 086-1106 Hokkaido Japan; 4 Department of Bacteriology II, National Institute of Infectious Diseases, 4-7-1 Gakuen, Musashimurayama-shi, Tokyo 208-0011 Japan

## Abstract

A multiple DNA inversion system, the shufflon, exists in incompatibility (Inc) I1 and I2 plasmids. The shufflon generates variants of the PilV protein, a minor component of the thin pilus. The shufflon is one of the most difficult regions for *de novo* genome assembly because of its structural diversity even in an isolated bacterial clone. We determined complete genome sequences, including those of IncI2 plasmids carrying *mcr-1*, of three *Escherichia coli* strains using single-molecule, real-time (SMRT) sequencing and Illumina sequencing. The sequences assembled using only SMRT sequencing contained misassembled regions in the shufflon. A hybrid analysis using SMRT and Illumina sequencing resolved the misassembled region and revealed that the three IncI2 plasmids, excluding the shufflon region, were highly conserved. Moreover, the abundance ratio of whole-shufflon structures could be determined by quantitative structural variation analysis of the SMRT data, suggesting that a remarkable heterogeneity of whole-shufflon structural variations exists in IncI2 plasmids. These findings indicate that remarkable rearrangement regions should be validated using both long-read and short-read sequencing data and that the structural variation of PilV in the shufflon might be closely related to phenotypic heterogeneity of plasmid-mediated transconjugation involved in horizontal gene transfer even in bacterial clonal populations.

## Introduction

The shufflon, one of the members of the site-specific recombination system, was discovered in 1986 in incompatibility (Inc) I1 plasmid R64^1, 2^. Shufflons have also been identified in IncI1γ^3^, IncI2^4, 5^, IncK^6, 7^ and IncZ plasmids^8, 9^. Shufflons generate variants of the PilV protein, a minor component of the thin pilus. The shufflon regions of R64 (IncI1), CoIIb-P9 (IncI1) and R721 (IncI2) plasmids consist of four (A, B, C and D), three (A, B and C) and three (A, BD and C) segments, respectively. Each segment, A, B, C and BD, includes two different partial open reading frames (ORFs) on the C-terminal region of PilV (ORF A, ORF A′; ORF B, ORF B′; ORF C, ORF C′; and ORF B′, ORF D′, respectively), whereas segment D only includes ORF D. These segments are rearranged in the conserved repeat region at the end of each segment by Rci, which has the activity of a site-specific tyrosine recombinase and is encoded by the *rci* gene^1, 10^. Komano *et al*. suggested that seven possible variants of *pilV* encode seven different PilV tip adhesins in IncI1 plasmid R64^3, 11^. PilV recognises specific lipopolysaccharide (LPS) structures on the surface of recipient cells during liquid mating^4, 12^. In particular, it has been reported that the ligands of the PilVA, PilVB′, PilVC and PilVC′ adhesins are the GlcNAc(β1–3)Glc, GlcNAc(α1–2)Glc, GlcNAc(β1–7)Hep and Glc(α1–2)Glc or Glc(α1–2)Gal structures, respectively, of LPS of *Escherichia coli* type R1 (*E*. *coli* O8), *E*. *coli* K-12 or *Salmonella enterica* Typhimurium LT2^6, 8, 13–15^. Thus, the shufflon rearrangement is closely related to plasmid transmission to a broad range of the *Enterobacteriaceae*.

Colistin has emerged as a treatment option for infectious diseases caused by carbapenemase-producing multidrug-resistant *Enterobacteriaceae* ^10, 11, 16–18^. In 2015, Liu *et al*. reported the emergence of a plasmid-mediated colistin resistance mechanism, MCR-1, in the *Enterobacteriaceae* ^11, 19, 20^. The *mcr-1* gene, which encodes phosphoethanolamine transferase MCR-1, was first identified in the pHNSHP45 plasmid, which is classified as an IncI2 plasmid, from the *E*. *coli* strain SHP45. In Japan, Suzuki *et al*. also identified five IncI2 plasmids carrying *mcr-1* in animal isolates^12, 18, 20, 21^. This gene has been reported in various plasmid types (namely IncF^13, 14, 22–25^, P^15, 26–28^, I2^11, 16–18, 29^, HI2^19, 20, 30^, repB (pO111)^31^, HI1^20^ and X4^18, 20, 21, 32^) of the *Enterobacteriaceae* with diverse origins worldwide, suggesting horizontal gene transfer and plasmid-mediated conjugal transfer of *mcr-1* among the *Enterobacteriaceae*. Therefore, analysis of plasmids carrying *mcr-1* has become important for the surveillance and control of drug-resistant bacteria.

Next-generation sequencers (NGSs) are powerful tools to reveal genomic features of organisms and are typically represented by SOLiD/Ion Torrent PGM from Life Sciences, Genome Analyzer/HiSeq 2000/MiSeq from Illumina and GS FLX Titanium/GS Junior from Roche^22–25, 33, 34^. As of September 2016, 76,793 records of genome sequences were submitted in the Assembly Database of the National Center for Biotechnology Information. Moreover, NGSs not only provide draft and complete genome sequences but are also used for quantitative analyses, including transcriptomic and metagenomic approaches^5, 26–28^. However, NGS short reads are too difficult to assemble because of frequent recombination regions, which include shufflon regions of bacterial plasmids^3, 29^. The DNA recombination-based pilus phase and antigenic variation systems have been found in several virulent bacteria (such as *Neisseria* spp., *Borrelia* spp., *Treponema pallidum* and *Mycoplasma* spp.)^30, 35^. These rearrangement regions are also difficult to assemble into a sequence structure. The introduction of the single-molecule, real-time (SMRT) sequencing, i.e. PacBio RS II (Pacific Biosciences), has dramatically changed the method for determination of bacterial complete genome sequences, and we have been able to easily obtain a gapless bacterial chromosomal sequence^32^. SMRT sequencing also enables uncovering long structural variations in human genomes^29, 33, 34^. However, little has been reported on quantitative structural variation analysis of isolated bacterial clones, even though it is known that several bacteria have DNA recombination-based variation systems.

In this study, we report the complete genome sequences, including those of IncI2 plasmids carrying *mcr-1*, of three *E*. *coli* strains, as well as quantitative structural variation and comparative analysis of whole-shufflon structures in the IncI2 plasmids.

## Results

### Genome sequencing and error corrections in shufflon regions of IncI2 plasmids

To determine the whole-genome sequences of colistin-resistant *E*. *coli* strains (MRY16-002 = 20Ec-P-124, MRY15-117 and MRY15-131), *de novo* assembly and circularisation were performed using SMRT sequencing data. The detailed *de novo* assembly results are summarised in Table S1. Sequences of IncI2 plasmids carrying *mcr-1* were detected in the PacBio assemblies of all strains. It has already been reported that the shufflon is a multiple DNA inversion system present in IncI2 plasmids^5, 36^ as described in the Introduction.

**Figure 1:**
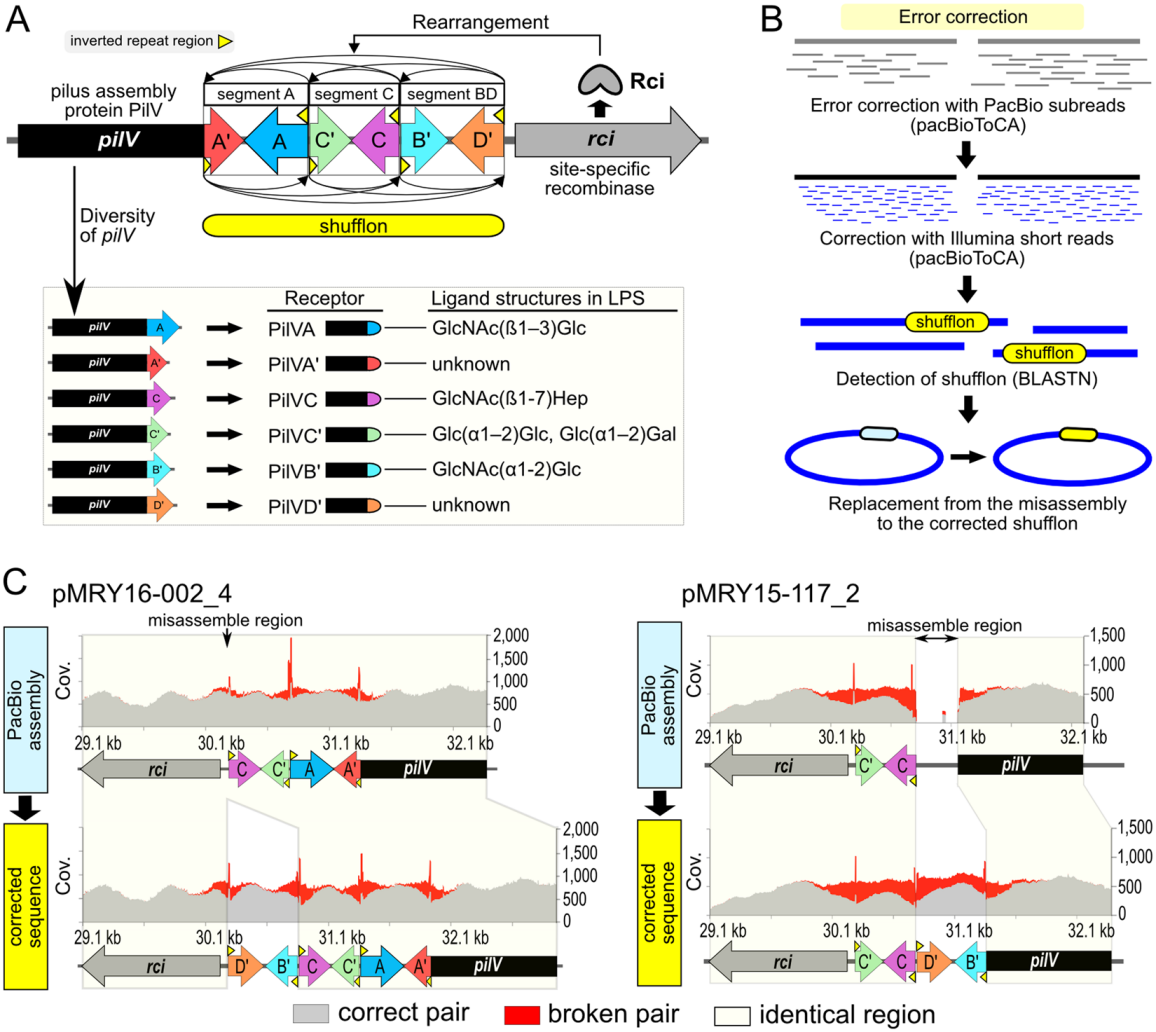
Error correction and validation of shufflons in IncI2 plasmids. (**A**) Schematic illustration of the model of shufflon rearrangement and relationship between PilV proteins and specific ligands. Site-specific recombination between any inverted repeat sequences, mediated by the site-specific recombinase Rci, yields the inversion of segments of a shufflon independently or among other segments. The various pilus PilV proteins recognise specific ligand structures in LPS. (**B**) Schematic representation of error correction in shufflon sequences. (**C**) Validations of shufflons using short-read mapping data. The X-axis and Y-axis represent the nucleotide positions of PacBio assemblies or corrected sequences and the coverage depth of the mapped short reads, respectively. Grey and red areas represent the coverage of the correct orientation and broken paired-end reads, respectively. Yellow boxes show identical regions between PacBio assemblies and corrected sequences.

Figure 1A shows the schematic illustration for the rearrangement of shufflons and relations between PilV proteins and specific ligand structures. Because the misassembling in a shufflon region might be caused by a heterogeneous population of shufflon structures in isolates, the error-corrected sequences of the shufflons were analysed by the pacBioToCA program with PacBio subreads and Illumina short reads as shown in Fig. 1B. The corrected sequences were composed of several combination patterns as follows: one segment (BD) in pMRY15–131_2; two segments (BD and C) in pMRY15-117_2; and three segments (A, B, D and C) in pMRY16-002_4. Moreover, the orientation and direction of the segments showed diversity of the detected shufflon structures. The corrected sequences carrying the most predominant shufflon structure were used in the subsequent analysis. The mapping validation using Illumina short reads was performed against the PacBio assemblies and corrected sequences, indicating broken orientations of paired-end reads intensively detected in the shufflon regions (Fig. 1C). In particular, unmapped and redundant coverage regions were detected in the PacBio assemblies of pMRY15-117_2 and pMRY16-002_4 (Fig. 1C), suggesting that these PacBio assemblies might have been misassembled in the shufflon regions. On the other hand, the mapping data of pMRY15-131_2 indicated a normal coverage (data not shown). The mapping validation against corrected sequences revealed that segment BD was deleted from the PacBio assemblies of pMRY15-117_2 and pMRY16-002_4 (Fig. 1C). In particular, segment BD in the PacBio assembly of pMRY15-117_2 was replaced by sequences that were nonhomologous to shufflons. Although broken paired-end reads were still detected in the shufflon regions of corrected sequences, the misassembled regions were resolved by error corrections, suggesting that the shufflon regions could not be simply completed by automatic *de novo* assembling because of the heterogeneous population of shufflon structures in the isolates.

### Quantitative structural variation analysis of shufflon regions

**Figure 2:**
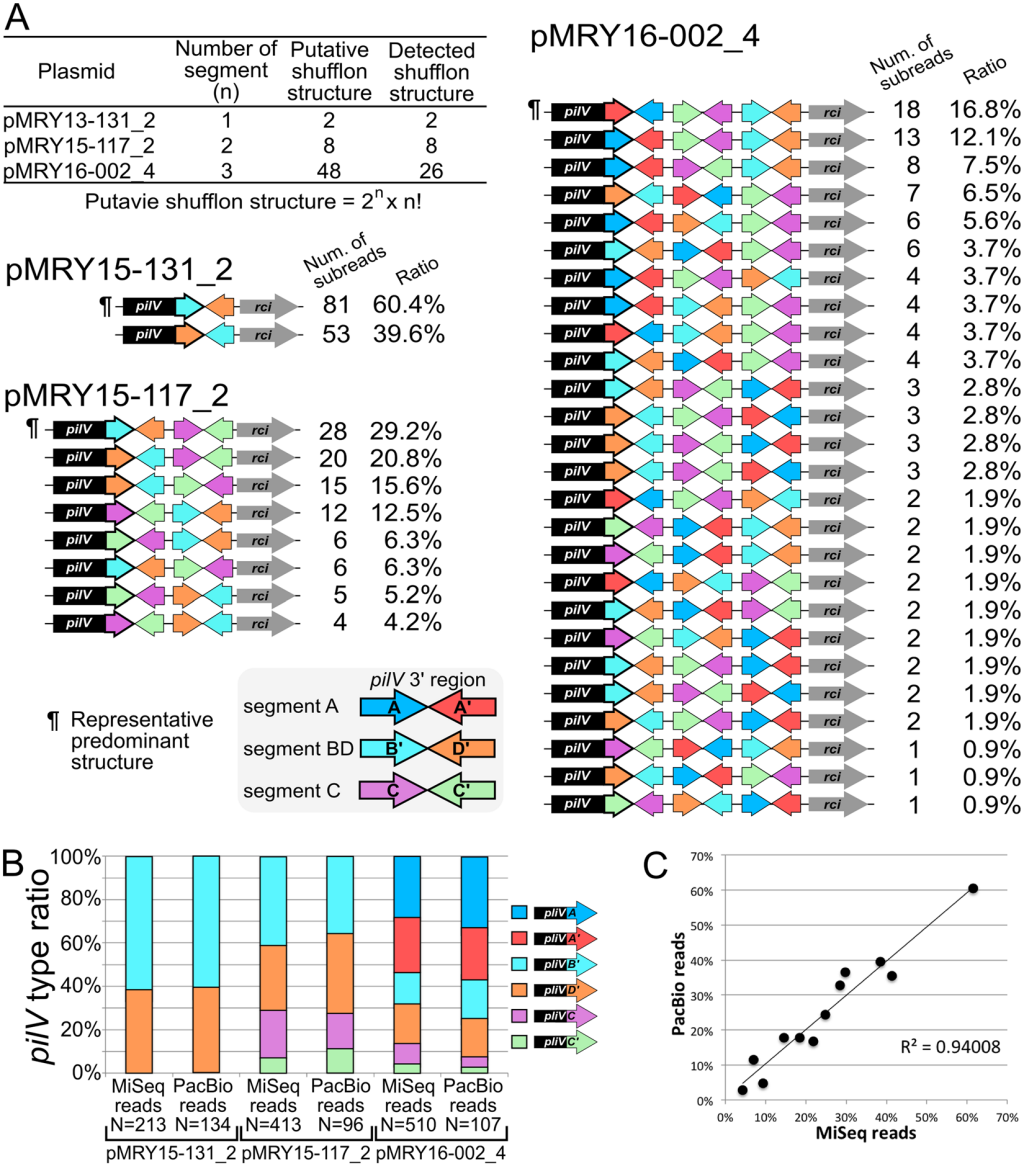
Quantitative structural variation analysis of shufflon regions in IncI2 plasmids. (**A**) Quantitative structural variation analysis of IncI2 plasmids. In pMRY13-131_2 and pMRY15-117_2, all shufflon structures were detected, whereas 26 shufflon structures were detected in pMRY16-002_4. (**B**) Comparison of the *pilV* gene ratios between different sequencer platforms. (**C**) Scatter plot of the *pilV* gene 3′ region ratios between the PacBio and MiSeq reads. The data indicate that there was a high correlation between the PacBio and MiSeq sequencer reads (R^2^ = 0.94).

The IncI2 plasmids with the corrected shufflon regions revealed that the number of shufflon segments was different among the three plasmids, as described above. It has been previously reported that rearrangements occur in shufflon segments and that possible variants of *pilV* can encode several different PilV tip adhesins^3, 37^. Our mapping validation data also showed the remarkable rearrangement of shufflon regions as shown in Fig. 1C. To reveal the abundance ratio of this rearrangement, quantitative structural variation analysis was performed as follows. Structural patterns for all shufflons were calculated according to the formula: number of shufflon structure combinations = 2^n^ × n! (where n = number of segments). This formula is suitable for only segments containing two 3′ ends of *pilV*, but not for segments containing one 3′ end of *pilV* (e.g. segment D as described for IncI1 shufflons). These numbers were 2, 8 and 48 for pMRY13-131_2, pMRY15-117_2 and pMRY16-002, respectively (Fig. 2A). All potential combinations of shufflon sequence structures were constructed, followed by actual shufflon structure detection using a BLASTN homology search with SMRT sequencing subreads. The quantitative structural variation analysis revealed that all potential combinations of structural variations were detected in pMRY13-131_2 and pMRY15-117_2. On the other hand, 26 combination structures were detected in the 48 potential combination patterns of the shufflon structures of pMRY16-002_4 (Fig. 2A). The quantitative analysis yielded the following results: (i) in pMRY15-131_2, two structures [i.e. gene loci *pilV*–ORF B′ (B′)–D′–*rci* and *pilV*–D′–B′–*rci*] were detected, and the *rci*–B′–D′–*pilV* pattern accounted for 60.4% of all patterns; (ii) in pMRY15-117_2, the *pilV*–B′–D′–C–C′–*rci*, *pilV*–D′–B′–C–C′–*rci*, *pilV*–D′–B′–C′–C–*rci*, *pilV*–C–C′–B′–D′–*rci*, *pilV*–C′–C–B′–D′–*rci*, *pilV*–B′–D′–C′–C–*rci*, *pilV*–C′–C–D′–B′–*rci* and *pilV*–C–C′–D′–B′–*rci* patterns accounted for 29.2%, 20.8%, 15.6%, 12.5%, 6.3%, 6.3%, 5.2% and 4.2%, respectively; (iii) in pMRY16-002_4, *pilV*–A′–A–C′–C–B′–D′–*rci*, *pilV*–A–A′–C′–C–B′–D′–*rci*, *pilV*–A–A′–C–C′–B′–D′–*rci* and other patterns accounted for 16.8%, 12.1%, 7.5% and 63.6%, respectively. Interestingly, the abundance ratio of shufflon structure patterns was uneven in two of the IncI2 plasmids, pMRY15-117_2 and pMRY16-002_4 (Fig. 2A). Variants of the PilV protein are generated by the rearrangement between the *pilV* 3′-end and a shufflon segment. The abundance ratio of *pilV* gene variants was also calculated on the basis of SMRT sequencing subreads and Illumina short reads using the junction between the *pilV* 3′-end and a shufflon segment (Fig. 2B). The quantitative analysis using short reads was valid because these reads included both the *pilV* 3′-end and shufflon segment regions. To validate the data of the quantitative structural variation analysis as described above, the abundance ratio of the *pilV* gene was compared between sequencing reads obtained using the two different platforms, i.e. SMRT sequencing subreads and Illumina short reads (Fig. 2C). These detected abundance ratios showed a high correlation between the two distinct platforms (R^2^ = 0.94), suggesting that the SMRT sequencing data is valid for the quantitative analysis of combination patterns not only of the *pilV* gene but also of whole-shufflon structures. The complete IncI2 plasmid sequences were determined using the predominant shufflon structures as follows: *pilV*–B′–D′–*rci* in pMRY15-131_2, *pilV*–B′–D′–C–C′–*rci* in pMRY15-117_2 and *pilV*–A′–A–C′–C–B′–D′–*rci* in pMRY16-002_4 (¶ in Fig. 2A).

### Genomic information of strains MRY16-002, MRY15-117 and MRY15-131

**Figure 3:**
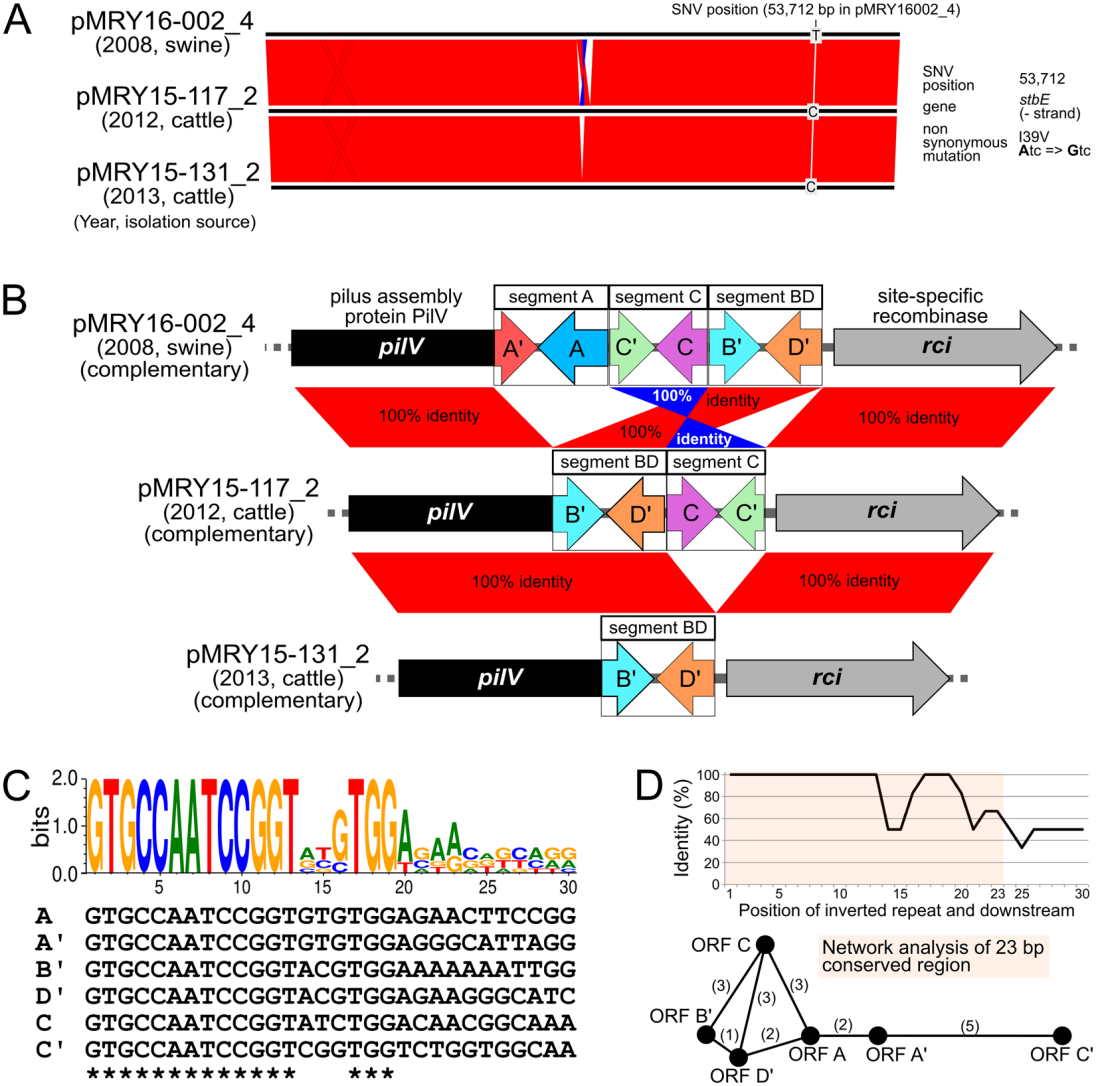
Comparative analysis of IncI2 plasmids pMRY13-131_2, pMRY15-117_2 and pMRY16-002_4. (**A**) Schematic comparative analysis of whole IncI2 plasmid sequences. The red and blue bars between chromosomal DNA sequences represent individual nucleotide matches in the forward and reverse directions, respectively. (**B**) Schematic representation and comparison of shufflon regions and flanking regions. Six coloured arrows (orange, sky blue, purple, light green, red and blue) represent the 3′ ORF region of the *pilV* gene in IncI2 plasmid shufflon segments. A grey arrow and black box represent the *rci* gene and main *pilV* gene, respectively. (**C**) Multiple alignments of six repeat regions of shufflon segments. These repeat regions were analysed by the sequence logo program. (**D**) Identical ratio plot of the 3′-end of shufflon regions and minimum spanning network analysis of SNVs in 23-bp conserved repeat regions. The numbers on the edges of the network show the number of SNVs between the repeat sequences.

The complete chromosomal and plasmid sequences were determined, and it was found that *E*. *coli* MRY16-002, MRY15-117 and MRY15-131 harboured six, two and two plasmids, respectively (Table 1). Two short plasmids, pMRY16-002_5 (6,078 bp) and pMRY16-002_6 (4,073 bp), were only detected using the Illumina data because the average length of PacBio long-read DNA library was approximately 30 kb. Multilocus sequence typing (MLST) analysis revealed that MRY16-002 and the other two strains (MRY15-117 and MRY15-131) belonged to two different sequence types (STs), ST117 and ST457, respectively. *In silico* serotype analysis showed different patterns in the three strains, and the chromosomal virulence gene profiles were also different between MRY15-117 and MRY15-131 (Table S2). However, the plasmid profiles indicated the same pattern for MRY15-117 and MRY15-131. Plasmid copy number analysis showed that the IncI2 plasmids carrying *mcr-1* were present in a single-copy per genome in each strain (Table 1). Comparative analysis indicated that the sequence structures were highly conserved among the three IncI2 plasmids. A single-nucleotide variation (SNV) was only detected in the *stbE* gene (nucleotide position 53,712 in pMRY16-002_2; Fig. 3A). Insertion/deletion sequences were only detected in the shufflon regions. All plasmids contained segment BD in the shufflon region, but segment A only existed in pMRY16-002_4 (Fig. 3B). The multiple alignments showed that 5′-GTGCCAATCCGGTNNGTGGA-3′ (20 bp) was a highly conserved sequence among the repeat regions in the shufflon segments (Fig. 3C and Table S3). The repeat sequences of each segment A and BD showed complete identity. However, those of segment C included three nucleotide variations, consistent with a previous report^29, 35^. A more detailed analysis showed that 23 bp at the ends of the shufflon segments were conserved, but variations from one to six nucleotides existed across these segment ends. The minimum spanning network analysis revealed low stability of repeat regions of ORF C′ (Fig. 3D and Table S3). The methylome analysis revealed the following results: (i) N^6^-Methyladenosine (m6A) methylation sites were found spread all over the genome; (ii) Dam methylation sequence pattern (i.e. 5′-GATC-3′) was detected most frequently; and (iii) unique methylation motifs were detected in each strain (Figure S1 and Table S4). However, the m6A methylation site was not detected in all shufflon inverted repeat regions (Figure S1).

**Table 1:** General features of three isolates and their complete genome sequences.

Strain name	Organism	Country	Isolation source	Year	*In silico* serotyping	Replicon	Chromosomal sequence type or plasmid Inc type	Drug resistance genes	Length (bp)	Average coverage^a^	Copy number^b^
MRY16-002 (=20Ec-P-124)	*E*. *coli*	Japan	swine	2008	O24:H4	chromosome	ST117	N.D.	4,920,828	135.41	1.0
						pMRY16-002_1	IncFIB and X1	N.D.	168,972	185.91	1.4
						pMRY16-002_2	N.D. (phage-like plasmid)	N.D.	108,986	298.92	2.2
						pMRY16-002_3	IncP	*aph*(*3*′)*-Ia*, *tet*(*A*)	108,957	176.26	1.3
						pMRY16-002_4	IncI2	*mcr-1*	61,805	219.75	1.6
						pMRY16-002_5	Col156	N.D.	6,078	N.A.	N.A.
						pMRY16-002_6	N.D.	N.D.	4,073	N.A.	N.A.
MRY15-117	*E*. *coli*	Japan	cattle	2012	O11:H25	chromosome	ST457	*sul2*, *strA*, *strB*, *tet*(*A*), *floR*	5,117,319	164.98	1.0
						pMRY15-117_1	IncFIB and FII	*dfrA14*, *mph*(*A*), *erm*(*B*), *aac*(*3*)*-IIa*, *∆bla*_TEM-1_, *bla*_CTX-M-27_	116,529	269.38	1.6
						pMRY15-117_2	IncI2	*mcr-1*	61,223	221.03	1.3
MRY15-131	*E*. *coli*	Japan	cattle	2013	O1:H25	chromosome	ST457	*sul2*, *strA*, *strB*, *tet*(*A*)	5,042,704	135.07	1.0
						pMRY15-131_1	IncFIB and FII	*dfrA14*, *mph*(*A*), *erm*(*B*), *aac*(*3*)*-IIa*, *∆bla*_TEM-1_, *bla*_CTX-M-27_	116,529	218.3	1.6
						pMRY15-131_2	IncI2	*mcr-1*	60,722	187.18	1.4

^a^The average coverage of complete sequences was calculated by PacBio raw-read mapping analysis using the SMRT portal software.

^b^The copy number was calculated by the number of plasmid coverage divided by that of chromosomal coverage.

ST, sequence type; bp, base pair; N.D., not detected; N.A., not available.

### Comparative analysis of the whole IncI2 plasmid sequences

**Figure 4:**
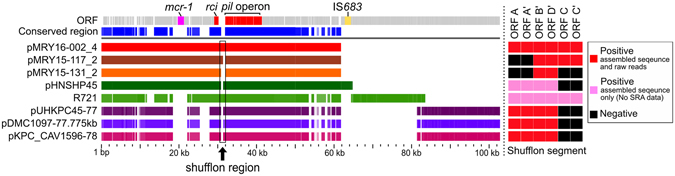
Comparative plasmid analysis of eight complete IncI2 plasmids. The area included shows that the percentage of identity between similar regions in the reference plasmid, pMRY16-002_4, and the other plasmids was at least 80%. The existence patterns of shufflon segments are shown on the right side.

To reveal the conservation of the whole IncI2 plasmid sequences, comparative analysis was performed for pMRY16-002_4, pMRY15-117_2, pMRY15-131_2 and the following five plasmids: (i) pUHKPC45-77, pDMC1097-77.775 kb and pKPC_CAV1596-78 using raw sequencing data deposited in the Short-Read Archive (SRA) database; (ii) pHNSHP45, a *mcr-1*-positive plasmid, using unregistered data in SRA; and (iii) R721, the first reported IncI2 plasmid, used as a reference. The length of the conserved region was 47,362 bp or 76.63% of the 61,805-bp sequence of pMRY16-002_4 (Fig. 4). The backbones of the IncI2 plasmids were divided into three types as follows: (i) pMRY15-131_2, pMRY15-117_2, pMRY16-002_4 and pHNSHP45; (ii) R721; and (iii) pUHKPC45-77, pDMC1097-77.775 kb and pKPC_CAV1596-78. The sequence structure of pHNSHP45 showed high similarity to those of three plasmids (pMRY15-131_2, pMRY15-117_2 and pMRY16-002_4); there were differences in the shufflon region and an insertion of a mobile element containing the IS*683* sequence (Fig. 4). The *rci* gene and the *pil* operon were conserved in all plasmids; conversely, the composition of the shufflon segments showed variability. Shufflon segments were detected using assembled sequences and NGS sequencing raw reads because of the reevaluation of the shufflons in the deposited complete IncI2 plasmid sequences. Segment BD was detected in all plasmids; however, segment C was only detected in pMRY15-117_2 and pMRY16-002_4 (Fig. 4). Although 18 complete IncI2 plasmid sequences are available in the GenBank database, NGS raw sequencing reads of only three of them were retrieved from the SRA database (Table S5). Among the other 15 IncI2 plasmids, whose sequences have only been deposited in GenBank, 12 plasmids harbour segments A and BD but not segment C. These results showed that segment BD shows a noticeable stability in the shufflon of IncI2 plasmids.

## Discussion

The SMRT sequencer and the hierarchical genome assembly process (HGAP) assembler are able to generate long reads and easily produce long assembled contigs without gap regions^5, 29, 32^. Chromosomal and plasmid sequences were assembled into one contig in this study. However, misassembled sequences were detected in the shufflon regions of IncI2 plasmids pMRY15-117_2 and pMRY16-002_4 because of high heterogeneity of these regions in the isolated clones. Brouwer *et al*. also discussed the utility of IncI1 plasmids and shufflon region sequences using Roche-454 and Illumina platforms^29, 35, 38, 39^. Although error correction programs for PacBio unitigs, iCORN2^6, 8, 36^ and Pilon^29, 37^ have been released, the heterogeneous remarkable rearrangement region could interfere with the correction software, preventing obtaining a correct complete sequence. Therefore, it is suggested that careful hybrid analysis using SMRT and short-read sequencing is necessary to resolve sequencing errors in the high rearrangement region. In contrast, the quantitative analysis of the *pilV* gene structure was performed using SMRT and Illumina sequencing data; however, the Illumina reads were too short to analyse the structural variation of the whole-shufflon sequence. The discordant quantitative analysis between SMRT and Illumina sequencing data might be caused by long single-nucleotide and/or tandem repeat regions that were difficult to sequence in Illumina data. Thus, it is strongly suggested that SMRT sequencing is used for the quantitative structural variation analysis.

It has previously been reported that the combination pattern of shufflon segments in IncI1 plasmids is conserved within the same STs^29, 40^. Our complete genome sequence analysis revealed that the IncI2 plasmids, excluding the shufflon region, were highly conserved among *E*. *coli* MRY16-002 (ST117), MRY15-117 (ST457) and MRY15-131 (ST457). In particular, the only difference between the IncI2 plasmids from the two ST457 strains was the insertion or deletion of segment C (Fig. 3B). However, the compositions of virulence factor genes were different between MRY15-117 and MRY15-131 (Table S2). Thus, it is suggested that the combination pattern of shufflon segments is variable within the same ST and among different STs, similar to transposable virulence factor genes, which constitute part of accessory genes.

The quantitative analysis of shufflon regions revealed that there are various shufflon structures in each strain (Fig. 2A). Previous studies have discussed the mechanism of shufflon rearrangement and the variability of shufflon sequences among IncI-type plasmids^5, 29, 41^. Our study is the first report on quantitative analysis of the whole-shufflon structure rearrangement in each isolated bacterial clone. Initially, we supposed that the abundance ratio in the shufflon structure was stochastically equal for each isolated clone. Indeed, the IncI2 plasmid pMRY15-131_2, which only has shufflon segment BD, showed nearly perfect evenness in the structural variation. In contrast, the ratios of structural variation showed deviations in pMRY16-002_4 and pMRY15-117_2, which possess two and three shufflon segments, respectively. In particular, the abundance ratios of *pilVC* and *pilVC′* were lower than those of other *pilV* genes in pMRY16-002_4 and pMRY15-117_2 (Fig. 2B). These deflections in recombination events might correlate with the types of inverted repeat sequences in the shufflon. It has previously been reported that the site-specific recombinase Rci promotes site-specific recombination between any two of 19-bp inverted repeat sequences of the shufflon^35, 38, 39, 42^. Our results showed that the nucleotide identity ranged from 85% to 100% among 20-bp repeat regions of the six segments; as shown above, the length of the conserved repeat region was up to 23 bp (Fig. 3D). In the 23-bp conserved region, the repeat sequences of ORF C′ showed low identify (approximately 74 to 78%) to other repeats; there were two-nucleotide, one-nucleotide and six-nucleotide differences in the 23-bp conserved region of segments A, BD and C, respectively. Hence, the deflection in the shufflon structural variation might be related to the nucleotide polymorphism in the 23-bp repeat regions, and the low stability of the repeat region in segment C might also lead to low abundance ratios of *pilVC* and *pilVC′*. Moreover, the plasmid copy number analysis showed that the IncI2 plasmids were single-copy plasmids (Table 1), suggesting that a single bacterial cell carries an IncI2 plasmid with a single *pilV* type. Taken together, these results imply that pili consisting of various PilV proteins are unevenly present in bacterial clonal populations even though individual bacterial cells may express a single PilV protein.

The methylation analysis revealed that m6A methylation sites were found spread all over the genome in three strains; however, these methylation sites were not detected in all shufflon inverted repeat regions (Figure S1 and Table S4). It was reported that the methylated nucleotides affect DNA-protein interaction, and m6A is involved in many biological phenomenon in bacteria (i.e. bacterial defence against bacteriophage, regulation of chromosome replication, mismatch repair, conjugal transfer of plasmids, packaging of phage DNA, and transcriptional regulation of virulence genes)^43^. Thus, it is suggested that m6A methylation does not directly influence the interaction between Rci recombinase and shufflon inverted repeat regions and rearrangement of the shufflon structure.

It has previously been reported that rearrangements in the shufflon determine the specificity of bacterial recipients^6, 8, 44^, i.e. that PilV structures specifically recognise the LPS pattern. Comparative analysis revealed that the four *mcr-1*-positive plasmids, i.e. pMRY15-131_2, pMRY15-117_2, pMRY16-002_4 and pHNSHP45, were highly conserved in all sequence regions, except the shufflon regions, which showed the presence of six, four and two PilV structures in pMRY16-002_4, pMRY15-117_2 and pMRY15-131_2, respectively (Fig. 3A). These results suggest that the IncI2 plasmid encoding six PilV structures, pMRY16-002_4, might have diverse transconjugation ability among the *Enterobacteriaceae*. Moreover, comparative analysis of the shufflon regions among 21 IncI2 plasmids revealed the deletion of segment C in 16 plasmids (Table S5). Brouwer *et al*. discussed that careful review of contigs produced by automated assembly may be needed^29, 45^, which is consistent with the results of this study. Among the 18 IncI2 plasmid sequences deposited in GenBank, only those of three plasmids (pUHKPC45-77, pDMC1097-77.775 kb and pKPC_CAV1596-78) were available in the SRA database (Table S5). The patterns of shufflon segments were determined using Illumina and/or PacBio raw reads in six plasmids (pMRY15-131_2, pMRY15-117_2, pMRY16-002_4, pUHKPC45-77, pDMC1097-77.775 kb and pKPC_CAV1596-78) and were the same as those in the assembled sequence data. Although there is a possibility that some misassembled sequence information has been deposited in public databases, segments C and BD of IncI2 plasmids might tend to carry insertions or deletions and have noticeable stability, respectively.

In conclusion, we demonstrated that SMRT sequencing is effective in quantitative structural variation analysis of regions with high rearrangement rates, including the shufflon. This analysis could reveal not only the diversity of shufflon structures but also the actual rearrangement status for the shufflon in isolated bacterial clones. The shufflon is important for IncI2 conjugative plasmid transfer in various *Enterobacteriaceae*. Moreover, detection of *mcr-1*-carrying IncI2 plasmids of diverse origin has been reported worldwide, and multidrug-resistant bacteria, including those resistant to colistin, have become a global issue. Thus, the detection of whole-shufflon structures and *pilV* gene variants might be effective for control and surveillance of antimicrobial-resistant bacteria harbouring IncI2 plasmids.

## Methods

### Bacterial strains and DNA extraction

Three strains of *E*. *coli* were used in the present study (Table 1). These single-clone strains were cultured in Luria–Bertani broth at 37 °C for 24 h under aerobic conditions. To prepare long-chain genomic DNA, cultured isolates were treated with 0.1% sodium dodecyl sulphate in TE buffer for 30 min at 65 °C, followed by proteinase K treatment for 4 h at 55 °C. Phenol/chloroform was used for removing proteins from crude DNA samples, followed by dialysis against TE buffer. The quality of the genomic DNA was analysed by 1% agarose gel electrophoresis with GelRed (Biotium, Inc., Hayward, CA, USA), and the purity of DNA was assessed using a NanoDrop spectrophotometer (NanoDrop Technologies). Plasmid isolation was performed by pulsed-field gel electrophoresis with S1 nuclease-treated agarose plugs. The gel-extracted plasmids were purified using the Wizard gel purification kit (Promega, Madison, WI, USA).

### Sequencing

Short-insert DNA libraries (approximately 400 bp in length) were constructed using a NexteraXT sample prep kit according to the manufacturer’s instructions (Illumina, Inc., San Diego, CA, USA), and the libraries were sequenced using MiSeq with the MiSeq version 3 600 cycle reagent kit (Illumina, Inc.). Genomic DNA was sheared by g-TUBE (Covaris, Inc., Woburn, MA, USA) followed by size selection using BluePippin (Sage Science, Inc., Beverly, MA, USA). Long-insert DNA libraries (approximately ≥20 kb in length) were constructed using the SMRTBell template prep kit, version 1.0, according to the manufacturer’s instructions (Pacific Biosciences, Menlo Park, CA, USA). The samples were sequenced using the PacBio RSII (Pacific Biosciences) with a movie length of 240 min; one SMRT cell was used for each sample.

### *De novo* assembly and gap closing

To remove the adapter and low-quality regions from Illumina short reads, short paired-end 300-mer reads were analysed by the Skewer software version 0.2^40, 46^ and Sickle version 1.33 (https://github.com/najoshi/sickle). The trimmed short reads were assembled using the A5 MiSeq software version 20140604^41, 47^. For adapter removal and *de novo* assembly with long reads, SMRT raw reads were analysed using RS HGAP Assembly in SMRT Analysis version 2.3. The error correction of long-subread sequences was also performed using pacBioToCA in Celera Assembler version 8.2^42, 48^ for SMRT subreads and quality-trimmed Illumina short reads. The assembled contigs of long subreads were manually removed at 5′-end and 3′-end low-coverage regions (≤×100 coverage). A BLASTN homology search^44, 49^ was performed against each prime-end region; the gap regions were closed at completely identical regions. In IncI2 plasmids, the shufflon region was replaced using the corrected long subreads. To detect incorrect gap closing and misassembled sequences, SMRT long subreads and Illumina short reads were aligned to tentative complete genomes using BLASR version 1^50^ and BWA-MEM^45, 51^, respectively.

### Annotation

Gene prediction was performed for complete genome sequences with the PROKKA version 1.11^46^, followed by InterProScan^47^ and BLASTP searches using the nr database for validation. Searches for drug resistance genes, virulence factor genes, plasmid Inc types and serotypes were performed using the ABRicate program version 0.2 (https://github.com/tseemann/abricate) with ResFinder^48^ and the Lahey β-lactamase database (https://www.ncbi.nlm.nih.gov/projects/pathogens/beta-lactamase-data-resources/), VirulenceFinder^49^ and the VFDB^51^ database, PlasmidFinder^52^ database and SerotypeFinder^53^ database, respectively. MLST of *E*. *coli* was performed by the MLST program version 1.2 (https://github.com/tseemann/mlst/issues) against the PubMLST typing database.

### Quantitative structural variation analysis

The putative structural variation of the shufflon was calculated with the number of detected shufflon segments in the corrected long subreads; all possible shufflon structural sequence patterns were constructed. To detect and calculate the real structural variation, these possible sequences were searched against PacBio subreads using BLASTN; the detected subreads were counted only if they contained adjacent shufflon regions of the *pilV* and *rci* genes. The recombined *pilV* sequence type was also analysed by BLASTN using the MiSeq-trimmed reads and counted with only short reads containing a part of the main *pilV* region, an inverted repeat sequence and a part of the shufflon segment.

### Methylome analysis

Detection of the N^6^-Methyladenosine (m6A) methylation site and methylated motif was performed by RS_Modification_and_Motif_Analysis protocol in the SMRT Analysis version 2.3 using the standard mapping protocol. The interpulse duration (IPD) ratio of detected methylation sites was visualised using Circos version 0.69^54^.

### Comparative plasmid analysis

A BLASTN homology search was performed for comparative sequence analysis of pMRY15-131_2, pMRY15-117_2 and pMRY16-002_4, followed by alignment visualisation with the ACT^55^ and Easyfig^56^ programs. SNVs in 23-bp repeat regions were compared by minimum spanning network analysis of PopART (http://popart.otago.ac.nz). Whole-plasmid comparative analysis was performed using the GView server with the default parameters (https://server.gview.ca/)^57^ for eight complete IncI2 plasmid sequences. The shufflon segments were searched by BLASTN against complete IncI2 plasmid sequences and NGS raw reads.

### Data deposition

Raw sequence reads were deposited in the DDBJ Sequence Read Archive under accession number DRA004888 (BioProject: PRJDB5007, BioSample: SAMD00056130–SAMD00056132 and Experiment: DRX060089–DRX060094). The complete genome sequences were deposited in DDBJ under the following accession numbers: chromosome of strain MRY16-002, AP017610; pMRY16-002_1, AP017611; pMRY16-002_2, AP017612; pMRY16-002_3, AP017613; pMRY16-002_4, AP017614; pMRY16-002_5, AP017615; pMRY16-002_6, AP017616; chromosome of strain MRY15-117, AP017617; pMRY15-117_1, AP017618; pMRY15-117_2, AP017619; chromosome of strain MRY15-131, AP017620; pMRY15-131_1, AP017621l; and pMRY15-131_2, AP017622.

## Electronic supplementary material

Supplementary Information
