# Supplementary material for: Elucidation of quantitative structural diversity of remarkable rearrangement regions, shufflons, in IncI2 plasmids
Source: Sci Rep. 2017 Apr 19;7:928. doi: 10.1038/s41598-017-01082-y (PMC5430464; doi:10.1038/s41598-017-01082-y)
Supplement: Supplementary file 1 — Supplementary Information [file 41598_2017_1082_MOESM1_ESM.pdf]

Supplementary Information

Title

Elucidation of quantitative structural diversity of remarkable rearrangement regions, shufflons, in IncI2 plasmids

Author

Tsuyoshi Sekizuka[1]*, Michiko Kawanishi[2], Mamoru Ohnishi[3], Ayaka Shima[4], Kengo Kato[1], Akifumi Yamashita[1], Mari Matsui[4], Satowa Suzuki[4], Makoto Kuroda[1]

Affiliation

[1]Pathogen Genomics Center, National Institute of Infectious Diseases, 1-23-1 Toyama, Shinjyuku-ku, Tokyo 162-8640, Japan
[2]Assay Division II, Bacterial Assay Section, National Veterinary Assay Laboratory, Ministry of Agriculture, Forestry and Fisheries, 1-15-1 Tokura Kokubunji, Tokyo 185-8511 Japan
[3]Ohnishi Laboratory of Veterinary Microbiology, Japan
[4]Department of Bacteriology II, National Institute of Infectious Diseases, Musashimurayama, Tokyo 208-0011, Japan

* Corresponding author

Dr. Tsuyoshi Sekizuka

Laboratory of Bacterial Genomics, Pathogen Genomic Center, National Institute of Infectious Diseases

1-23-1 Toyama, Shinjuku-ku, Tokyo 162-8640, Japan

Tel: +[81]-3-5285-1111 ext. 2525

Fax: +[81]-3-5285-1166

E-mail: sekizuka@nih.go.jp

Supplementary material

Table S1. Summary of PacBio RSII run and HGAP3 assembly information

Section	Protocol / Job Metric		Sample name		
			MRY16-002	MRY15-117	MRY15-131
Methods	PacBio RSII run status	Average length of Input DNA library (bp)	31,247	34,876	27,218
		Number of SMRT Cells	1	1	1
		Movie length (min)	240	240	240
	HGAP3 Filterring paramter	Minimum Subread Length	500	500	500
		Minimum Polymerase Read Quality	0.8	0.8	0.8
		Minimum Polymerase Read Length	100	100	100
	HGAP3 assembly paramter	Minimum Seed Read Length	6,000	6,000	6,000
		Number of Seed Read Chunks	6	6	6
		Alignment Candidates Per Chunk	10	10	10
		Total Alignment Candidates	24	24	24
		Minimum Coverage For Correction	6	6	6
		Genome Size (Bp)	5,000,000	5,000,000	5,000,000
		Target Coverage	25	25	25
		Overlapper Error Rate	0.06	0.06	0.06
		Overlapper Min Length	40	40	40
		Overlapper K-mer	40	40	40
	HGAP3 mapping paramter	Maximum Divergence (%)	30	30	30
		Minimum Anchor size	12	12	12
Results of HGAP3	Filtering (Post-Filter)	Polymerase Read Bases	900,108,489	1,194,986,182	907,755,639
		Polymerase Reads	62,740	80,504	61,608
		Polymerase Read N50	19,893	20,216	20,230
		Polymerase Read Length	14,346	14,843	14,734
		Polymerase Read Quality	0.857	0.852	0.853
	Subread Filtering	Mean Subread length	11,893	12,011	12,205
		N50	17,237	16,717	17,679
		Total Number of Bases	898,815,238	1,193,475,427	906,550,170
		Number of Reads	75,575	99,357	74,273
	Adapters	Adapter Dimers (0-10bp)	0.01%	0.01%	0.00%
		Short Inserts (11-100bp)	0.00%	0.00%	0.00%
	Mapping	Mapped Subread Length N50 (bp)	16,483	14,897	16,037
		Mapped Polymerase Read Length 95% (bp)	28,320	28,360	26,850
		Mapped Subread Length Mean (bp)	10,855	10,109	10,399
		Mapped Polymerase Read Length Max (bp)	44,886	41,773	37,369
		Mapped Read	59,358	73,857	57,446

	Mapped Polymerase Read Length	12,992	12,238	12,455
	Mapped Polymerase Read Length n50	18,758	17,843	18,060
	Mapped Subreads	70,846	88,973	68,599
	Mapped Subread Bases	769,056,256	899,432,250	713,389,598
	Mapped Subread Length	10,855	10,109	10,399
	Mean Mapped Subread Concordance	0.863	0.849	0.857
Coverage	Mean Coverage	135.51	159.11	132.57
	Missing Bases (%)	0.04	0.2	0
Pre-Assembler Report	Polymerase Read Bases	898,815,238	1193475427	906550170
	Length Cutoff	23,221	24,800	23,363
	Seed Bases	150,001,201	150,016,858	150,016,570
	Pre-Assembled bases	107,431,279	80,209,190	80,373,405
	Pre-Assembled Yield	0.716	0.535	0.536
	Pre-Assembled Reads	6,108	6,059	5,995
	Pre-Assembled Reads Length	17,588	13,238	13,406
	Pre-Assembled N50	23,981	24,568	23,716
Polished Assembly	Polished Contigs	5	6	3
	Max Contig Length	4,942,963	5,144,467	5,062,492
	N50 Contig Length	4,942,963	5,144,467	5,062,492
	Sum of Contig Lengths	5,497,902	5428266	5297069
	High coverage Contigs (≥ x100)	5	3	3

Table S2. Comparison of drug resistance and virulence factor genes among three isolates

Category	Gene	Product	MRY16-002		MRY15-117		MRY15-131	
			Chromosome	Plasmid	Chromosome	Plasmid	Chromosome	Plasmid
Drug resistance	*sul2*	sulfonamide-resistant dihydropteroate synthase Sul2	-	-	+	-	+	-
	strA	streptomycin phosphotransferase StrA	-	-	+	-	+	-
	strB	streptomycin phosphotransferase StrB	-	-	+	-	+	-
	tet(A)	tetracycline resistance protein TetA	-	+	+	-	+	-
	floR	florfenicol/ chloramphenicol export protein FloR	-	-	+	-	+	-
	dfrA14	dihydrofolate reductase DfrA14	-	-	-	+	-	+
	mph(A)	macrolide 2'-phosphotransferase	-	-	-	+	-	+
	erm(B)	rRNA adenine N-6-methyltransferase	-	-	-	+	-	+
	aac(3)-IIa	aminoglycoside N(3')-acetyltransferase	-	-	-	+	-	+
	bla$_{\text{CTX-M-27}}$	beta-lactamase CTX-M-27 precursor	-	-	-	+	-	+
	mcr-1	phosphoethanolamine transferase	-	+	-	+	-	+
	aph(3')-Ia	aminoglycoside 3'-phosphotransferase	-	+	-	-	-	-
Virulence factor	*afaA-VIII*	AfaA-VIII protein	-	-	-	-	+	-
	afaB-VIII	chaperone protein AfaB-VIII	-	-	-	-	+	-
	afaC-VIII	AfaC-VIII usher protein	-	-	-	-	+	-
	afaD-VIII	AfaD-VIII protein	-	-	-	-	+	-
	afaE-VIII	AfaE-VIII adhesin	-	-	-	-	+	-
	aslA	putative arylsulfatase	+	-	+	-	+	-
	cdtA	cytolethal distending toxin subunit A precursor	+	-	-	-	-	-
	cdtB	cytolethal distending toxin subunit B precursor	+	-	-	-	-	-
	cdtC	cytolethal distending toxin subunit C precursor	+	-	-	-	-	-
	chuA	outer membrane heme/hemoglobin receptor ChuA	+	-	+	-	+	-
	chuS	heme oxygenase ChuS	+	-	+	-	+	-
	chuT	periplasmic heme-binding protein ChuT	+	-	+	-	+	-
	chuU	heme permease protein ChuU	+	-	+	-	+	-
	chuV	ATP-binding hydrophilic protein ChuV	+	-	+	-	+	-
	chuW	putative oxygen independent coproporphyrinogen III oxidase	+	-	+	-	+	-
	chuX	putative heme-binding protein ChuX	+	-	+	-	+	-
	chuY	ChuY protein	+	-	+	-	+	-
	ecpA	pilus structural protein subunit EcpA	+	-	+	-	+	-
	ecpB	pilus chaperone protein EcpB	+	-	+	-	+	-
	ecpC	pilus usher protein EcpC	+	-	+	-	+	-
	ecpD	polymerized tip adhesin	+	-	+	-	+	-
	ecpE	pilus chaperone protein EcpE	+	-	+	-	+	-
	ecpR	regulator protein EcpR	+	-	+	-	+	-
	entA	2,3-dihydro-2,3-dihydroxybenzoate dehydrogenase	+	-	+	-	+	-
	entB	isochorismatase	+	-	+	-	+	-
	entC	isochorismate synthase 1	+	-	+	-	+	-
	entD	phosphopantetheinyl transferase component	+	-	-	-	-	-
	entE	2,3-dihydroxybenzoate-AMP ligase component	+	-	+	-	+	-
	entF	enterobactin synthase multienzyme complex component	+	-	+	-	+	-
	entS	enterobactin exporter	+	-	+	-	+	-
	espL1	type III secretion system effector EspL1	+	-	+	-	-	-
	espR1	type III secretion system effector EspR1	-	-	+	-	+	-
	espX4	type III secretion system effector EspX4	-	-	+	-	+	-
	espY2	type III secretion system effector EspY2	-	-	+	-	+	-
	fepA	ferrienterobactin outer membrane transporter	+	-	+	-	+	-
	fepB	ferrienterobactin ABC transporter periplasmic binding protein	+	-	+	-	+	-
	fepC	ferrienterobactin ABC transporter ATPase	+	-	+	-	+	-
	fepD	ferrienterobactin ABC transporter permease	+	-	+	-	+	-
	fepG	iron-enterobactin ABC transporter permease	+	-	+	-	+	-
	fes	enterobactin/ferric enterobactin esterase	+	-	+	-	+	-
	fimA	type I fimbriae major pilin FimA	+	-	+	-	+	-
	fimB	type I fimbriae chaperone protein FimB	+	-	+	-	+	-
	fimC	type I fimbriae outer membrane usher protein precursor FimC	+	-	+	-	+	-
	fimD	type I fimbriae fimbrial adhesin FimD	+	-	+	-	+	-
	fimE	type I fimbriae regulatory protein FimE	+	-	+	-	+	-
	fimF	type I fimbriae adaptor protein FimF	+	-	+	-	+	-
	fimG	type I fimbriae FimG protein precursor	+	-	+	-	+	-
	fimH	type I fimbriae minor fimbrial subunit FimH, adhesin	+	-	+	-	+	-
	fimI	type I fimbriae fimbrial protein internal segment FimI	+	-	+	-	+	-
	gad	glutamate decarboxylase	+	-	+	-	+	-
	gspC	general secretion pathway protein C	+	-	+	-	+	-
	gspD	general secretion pathway protein D	+	-	+	-	+	-
	gspE	general secretion pathway protein E	+	-	+	-	+	-
	gspF	general secretion pathway protein F	+	-	+	-	+	-
	gspG	general secretion pathway protein G	+	-	+	-	+	-
	gspH	general secretion pathway protein H	+	-	+	-	+	-
	gspI	general secretion pathway protein I	+	-	+	-	+	-
	gspJ	general secretion pathway protein J	+	-	+	-	+	-
	gspK	general secretion pathway protein K	+	-	+	-	+	-
	gspL	general secretion pathway protein L	+	-	+	-	+	-

gspM	general secretion pathway protein M	+	-	+	-	+	-
ireA	siderophore receptor	+	-	+	-	+	-
irp1	yersiniabactin biosynthetic protein Irp1	+	-	-	-	-	-
irp2	yersiniabactin biosynthetic protein Irp2	+	-	-	-	-	-
irp3	yersiniabactin biosynthetic protein Irp3	+	-	-	-	-	-
irp4	yersiniabactin biosynthetic protein Irp4	+	-	-	-	-	-
irp5	yersiniabactin siderophore biosynthetic protein Irp5	+	-	-	-	-	-
irp6	lipoprotein inner membrane ABC-transporter Irp6	+	-	-	-	-	-
irp7	inner membrane ABC-transporter Irp7	+	-	-	-	-	-
irp8	putative signal transducer Irp8	+	-	-	-	-	-
irp9	salicylate synthase Irp9	+	-	-	-	-	-
fyuA	yersiniabactin receptor protein	+	-	-	-	-	-
iss	increased serum survival protein	+	+	+	-	+	-
kpsD	KpsD protein	-	-	+	-	+	-
lpfA	long polar fimbria protein LpfA	+	-	+	-	+	-
mchF	ABC transporter protein MchF	-	-	+	-	+	-
ompA	outer membrane protein A	+	-	+	-	+	-
papB	regulatory protein PapB	-	-	+	-	+	-
papC	usher protein PapC	-	-	+	-	-	-
papD	chaperone protein PapD	-	-	+	-	-	-
papH	P pilus termination subunit PapH	-	-	+	-	-	-
papI	regulatory protein PapI	-	-	+	-	+	-
papJ	P pilus assembly protein PapJ	-	-	+	-	-	-
papK	P pilus minor subunit PapK	-	-	+	-	-	-
papX	flagellum synthesis regulation protein PapX	-	-	+	-	+	-
pic	serine protease autotransporter Pic	+	-	-	-	-	-
tsh	haemoglobin protease	+	-	-	-	-	-
ybtA	yersiniabactin-related transcriptional regulator YbtA	+	-	-	-	-	-
iroB	glucosyltransferase IroB	-	+	-	-	-	-
iroC	ATP binding cassette transporter IroC	-	+	-	-	-	-
iroD	esterase IroD	-	+	-	-	-	-
iroE	esterase IroE	-	+	-	-	-	-
iroN	salmochelin receptor IroN	-	+	-	-	-	-
iucA	aerobactin synthesis protein IucA	-	+	-	-	-	-
iucB	aerobactin synthesis protein IucB	-	+	-	-	-	-
iucC	aerobactin synthesis protein IucC	-	+	-	-	-	-
iucD	aerobactin synthesis protein IucC, lysine 6-monooxygenase	-	+	-	-	-	-
katP	plasmid-encoded catalase peroxidase	-	+	-	-	-	-

+, presence; -, absence.

Table S3. Summary of the differences in 20 or 23 bp conserved region among shufflon inverted repeat regions

Number of mismatch in 20 bp conserved region (idenitiy)		Number of mismatch in 23 bp conserved region (idenitiy)					
		ORF A	ORF A'	ORF B'	ORF D'	ORF C	ORF C'
	ORF A		2 (91.3%)	3 (87.0%)	2 (91.3%)	3 (87.0%)	6 (73.9%)
	ORF A'	0 (100%)		5 (78.3%)	4 (82.6%)	5 (78.3%)	5 (78.3%)
	ORF B'	2 (90.0%)	2 (90.0%)		1 (95.7%)	3 (87.0%)	6 (73.9%)
	ORF D'	2 (90.0%)	2 (90.0%)	0 (100%)		3 (87.0%)	6 (73.9%)
	ORF C	2 (90.0%)	2 (90.0%)	2 (90.0%)	2 (90.0%)		6 (73.9%)
	ORF C'	3 (85.0%)	3 (85.0%)	3 (85.0%)	3 (85.0%)	4 (80.0%)	

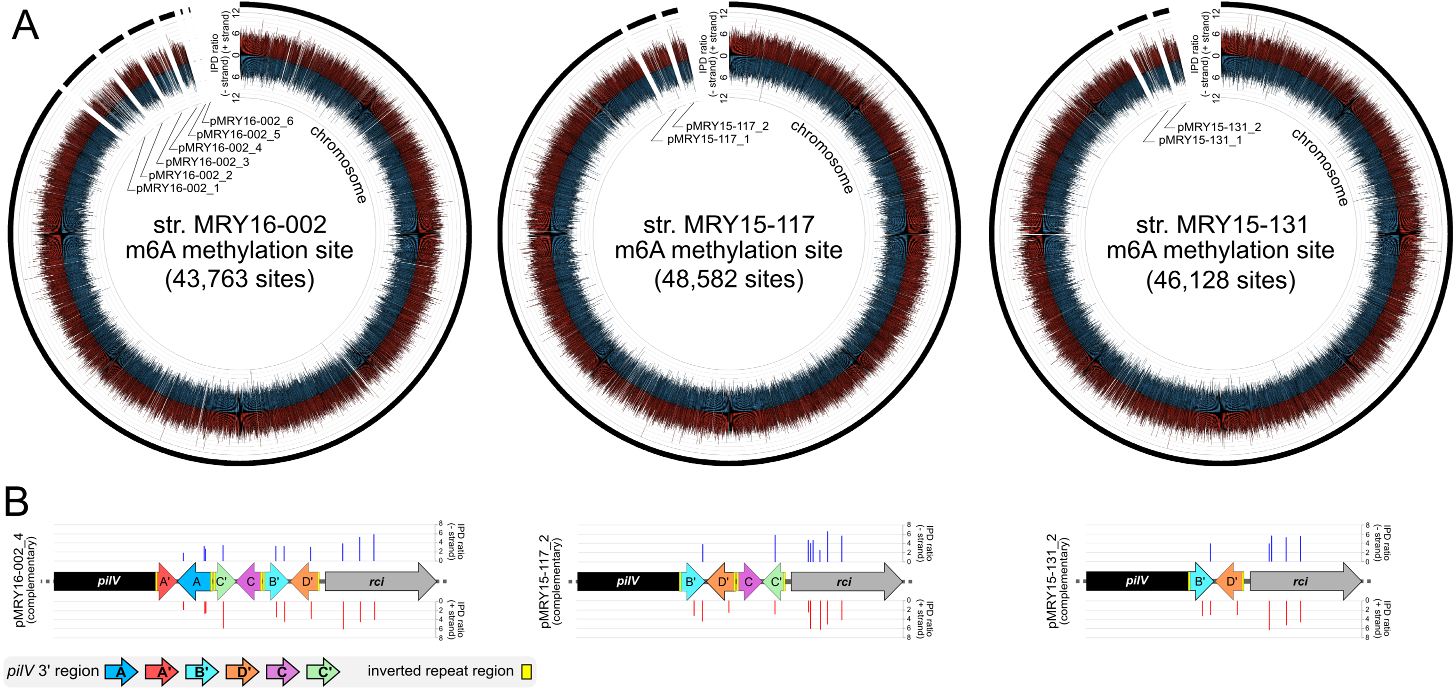

Figure S1 N^{6}-Methyladenosine (m6A) methylation site detected in three *Escherichia coli* strains.
(A) Circos plot displaying the genome-wide detections of m6A methylation site. The interpulse duration (IPD) ratio on the positive and negative strands are plotted as the red and blue bars, respectively. (B) Schematic representation of m6A methylation sites on shufflon regions and adjacent gene loci in three *E. coli* strains. The m6A methylation site is not detected in all shufflon inverted repeat regions.

Table S4. Summary of discovered methylation motifs in three *Escherichia coli* strains

			MRY16-002				MRY15-117				MRY15-131			
Motif	Modified Position	Modification Type	% Detected methylations (# detected methylations)	Mean Score	Mean IPD Ratio	Mean Coverage	% Detected methylations (# detected methylations)	Mean Score	Mean IPD Ratio	Mean Coverage	% Detected methylations (# detected methylations)	Mean Score	Mean IPD Ratio	Mean Coverage
G**A**TC	2	m6A	98.96% (42,145)	103.63	4.65	67.16	99.16% (41,899)	119.00	4.60	79.88	99.04% (41,499)	100.73	4.61	64.67
G**A**GNNNNRTAC	2	m6A	98.67% (519)	106.13	6.01	67.31	99.43% (521)	120.11	5.88	79.32	99.42% (512)	101.63	5.94	64.34
GT**A**YNNNNCTC	3	m6A	99.24% (522)	102.56	5.25	68.54	99.43% (521)	117.29	5.11	81.10	99.42% (512)	97.99	5.17	65.65
C**A**CNNNNNGGG	2	m6A	N.D.	N.D.	N.D.	N.D.	99.10% (1,535)	106.89	4.23	79.83	98.28% (1,488)	90.39	4.28	64.67
CT**A**NNNNNNTATC	3	m6A	98.63% (289)	100.26	5.16	69.59	N.D.	N.D.	N.D.	N.D.	N.D.	N.D.	N.D.	N.D.
GAT**A**NNNNNNTAG	4	m6A	98.29% (288)	101.05	5.49	69.42	N.D.	N.D.	N.D.	N.D.	N.D.	N.D.	N.D.	N.D.
AT**A**YNNNNNGTG	3	m6A	N.D.	N.D.	N.D.	N.D.	89.11% (1,039)	95.62	3.82	80.13	N.D.	N.D.	N.D.	N.D.
C**A**CNNNNNRTAT	2	m6A	N.D.	N.D.	N.D.	N.D.	99.57% (1,161)	114.81	4.73	80.79	N.D.	N.D.	N.D.	N.D.
GA**A**GNNNNNNTCA	3	m6A	N.D.	N.D.	N.D.	N.D.	99.58% (957)	116.52	5.72	78.10	N.D.	N.D.	N.D.	N.D.
TG**A**NNNNNNCTTC	3	m6A	N.D.	N.D.	N.D.	N.D.	98.75% (949)	118.23	4.81	80.54	N.D.	N.D.	N.D.	N.D.
AA**A**GNNNNNNTCA	3	m6A	N.D.	N.D.	N.D.	N.D.	N.D.	N.D.	N.D.	N.D.	99.91% (1,062)	97.79	6.19	62.07
TG**A**NNNNNNCTTT	3	m6A	N.D.	N.D.	N.D.	N.D.	N.D.	N.D.	N.D.	N.D.	99.25% (1,055)	100.16	5.07	65.90

N.D., Not detected.

Table S5. NGS platform information of complete IncI2 plasmids

Organim	Complete plasmid name	NGS platfrom	Sequence Read Archive (SRA) ID	Sequence accession number	Ref. (PMID)	PilV 3' end region serch with assembled sequences						PilV 3' end region serch witih NGS raw reads					
						ORF A	ORF A'	ORF B'	ORF D'	ORF C	ORF C'	ORF A	ORF A'	ORF B'	ORF D'	ORF C	ORF C'
Escherichia coli	pMRY15-131_2	Illumina MiSeq, PacBio	DRR065948, DRR065951	AP017622	this study	-	-	+	+	-	-	-	-	+	+	-	-
Escherichia coli	pMRY15-117_2	Illumina MiSeq, PacBio	DRR065947, DRR065950	AP017619	this study	-	-	+	+	+	+	-	-	+	+	+	+
Escherichia coli	pMRY16-002_4	Illumina MiSeq, PacBio	DRR065946, DRR065949	AP017614	this study	+	+	+	+	+	+	+	+	+	+	+	+
Escherichia coli	pHNSHP45	Illumina MiSeq	N.A.	KP347127	26603172	+	+	+	+	-	-	N.A.	N.A.	N.A.	N.A.	N.A.	N.A.
Salmonella enterica	pSH146_65	454 GS-FLX	N.A.	JN983044	23896467	+	+	+	+	+	pseudo	N.A.	N.A.	N.A.	N.A.	N.A.	N.A.
Escherichia coli	unnamed 3	PacBio	N.A.	CP009581	N.A.	+	+	+	pseudo	+	+	N.A.	N.A.	N.A.	N.A.	N.A.	N.A.
Escherichia coli	R721	Sanger	N.A.	AP002527	1400257	+	+	+	+	+	+	N.A.	N.A.	N.A.	N.A.	N.A.	N.A.
Escherichia coli	pChi7122-3	N.A.	N.A.	FR851304	22238616	+	+	+	+	-	-	N.A.	N.A.	N.A.	N.A.	N.A.	N.A.
Escherichia coli	pRM12761	Sanger, 454 GS-FLX, Illumina, PacBio	N.A.	CP007134	24855308	+	+	+	+	-	-	N.A.	N.A.	N.A.	N.A.	N.A.	N.A.
Escherichia coli	pRM13516	454 GS-FLX, Illumina HiSeq, PacBio	N.A.	CP006264	24410921	+	+	+	+	-	-	N.A.	N.A.	N.A.	N.A.	N.A.	N.A.
Escherichia coli	pHN1122-1	Sanger	N.A.	JN797501	23478963	+	+	+	+	-	-	N.A.	N.A.	N.A.	N.A.	N.A.	N.A.
Escherichia coli	pHNY2	454 GS-FLX	N.A.	KF601686	25987615	+	+	+	+	-	-	N.A.	N.A.	N.A.	N.A.	N.A.	N.A.
Escherichia coli	pCTXM132_P0421	IonTorrent	N.A.	KP198615	25861872	pseudo	pseudo	+	+	-	-	N.A.	N.A.	N.A.	N.A.	N.A.	N.A.
Escherichia coli	pHNLDH19	454 GS-FLX	N.A.	KM207012	25987615	+	+	+	+	-	-	N.A.	N.A.	N.A.	N.A.	N.A.	N.A.
Escherichia coli	pHNAH46-1	454 GS-FLX	N.A.	KJ020576	25987615	+	+	+	+	-	-	N.A.	N.A.	N.A.	N.A.	N.A.	N.A.
Escherichia coli	pCTXM64_C0967	IonTorrent	N.A.	KP091735	25861872	+	+	+	+	-	-	N.A.	N.A.	N.A.	N.A.	N.A.	N.A.
Shigella sonnei	p1081-CTXM	N.A.	N.A.	KJ460501	25405884	+	+	+	+	-	-	N.A.	N.A.	N.A.	N.A.	N.A.	N.A.
Klebsiella pneumoniae	pUHKPC45-77	PacBio, Illumina HiSeq	SRX283875, SRX277000	JMSX01000003	N.A.	+	+	+	+	-	-	+	+	+	+	-	-
Klebsiella pneumoniae	pBK15692	454 GS-FLX	N.A.	KC845573	23896467	+	+	+	+	-	-	N.A.	N.A.	N.A.	N.A.	N.A.	N.A.
Klebsiella pneumoniae	pDMC1097-77.775kb	PacBio, Illumina HiSeq	SRX276936, SRX305269, SRX1075361-4	CP011978	N.A.	+	+	+	+	-	-	+	+	+	+	-	-
Klebsiella pneumoniae	pKPC_CAV1596-78	HiSeq	SRR1582868	CP011645	25561339	+	+	+	+	-	-	+	+	+	+	-	-

N.A., not available; +, presence; -, absence; pseudo, pseudogene.
